# Missed postoperative metabolic acidosis associated with sodium-glucose transporter 2 inhibitors in cardiac surgery patients: a retrospective analysis

**DOI:** 10.1038/s41598-024-58853-7

**Published:** 2024-04-06

**Authors:** Hyeon A Kim, Joo Yeon Kim, Young Hwan Kim, Young Tak Lee, Pyo Won Park

**Affiliations:** 1 Department of Cardiovascular and Thoracic Surgery, Ewha Womans University Medical Center, Seoul, Republic of Korea; 2 Department of Cardiovascular and Thoracic Surgery, Incheon Sejong Hospital, Incheon, Republic of Korea

**Keywords:** Cardiology, Diseases, Risk factors

## Abstract

The increasing use of sodium glucose transporter 2 inhibitors (SGLT2i) for treating cardiovascular (CV) diseases and type 2 diabetes (T2D) is accompanied by a rise in euglycemic diabetic ketoacidosis occurrences in cardiac surgery patients. Patients undergoing cardiac surgery, due to their pre-existing CV disease which often requires SGLT2i prescriptions, face an increased risk of postoperative metabolic acidosis (MA) or ketoacidosis (KA) associated with SGLT2i, compounded by fasting and surgical stress. The primary aim of this study is to quantify the incidence of SGLT2i-related postoperative MA or KA and to identify related risk factors. We analyzed data retrospectively of 823 cardiac surgery patients, including 46 treated with SGLT2i from November 2019 to October 2022. Among 46 final cohorts treated preoperatively with SGLT2i, 29 (63%) developed postoperative metabolic complications. Of these 46 patients, stratified into two categories based on postoperative laboratory findings, risk factor analysis were conducted and compared. Analysis indicated a prescription duration over one week significantly elevated the risk of complications (Unadjusted OR, 11.7; p = 0.032*; Adjusted OR, 31.58; p = 0.014*). A subgroup analysis showed that a cardiopulmonary bypass duration of 60 min or less significantly raises the risk of SGLT2i-related postoperative MA in patients with a sufficient prescription duration. We omitted the term "diabetes" in describing complications related to SGLT2i, as these issues are not exclusive to T2D patients. Awareness of SGLT2i-related postoperative MA or KA can help clinicians distinguish between non-life-threatening conditions and severe causes, thereby preventing unnecessary tests and ensuring best practice.

## Introduction

The U.S. Food and Drug Administration (FDA) granted approval for dapagliflozin, a sodium-glucose transporter 2 inhibitor (SGLT2i), in 2011 for the treatment of type 2 diabetes (T2D). Subsequent research has extensively shown the efficacy of these inhibitors in treating a broader spectrum of cardiovascular (CV) diseases. Two landmark trials—DAPA-HF (Dapagliflozin in Patients with Heart Failure and Reduced Ejection Fraction)^1^ and EMPEROR-Reduced (Cardiovascular and Renal Outcomes with Empagliflozin in Heart Failure)^2^—have explored the effectiveness of SGLT2i in treating heart failure with reduced ejection fraction (HFrEF), irrespective of the presence of T2D. Further studies like EMPEROR-preserved (Empagliflozin in Heart Failure with a Preserved Ejection Fraction)^3^ and DELIVER (Dapagliflozin in Heart Failure with Mildly Reduced or Preserved Ejection Fraction)^4^ have broadened their applicability to HF patients with preserved or mildly reduced EF (HFpEF or HFmrEF) irrespective of T2D status. In alignment with these findings, the 2021 European Society of Cardiology guidelines for the treatment of acute and chronic HF recommended dapagliflozin or empagliflozin for patients with HFrEF to reduce the risk of hospitalization for HF (HHF) and death (Class IA)^5^. Furthermore, the 2022 ACC/AHA/HFSA Guideline for the Management of HF^6^ advocated for the use of SGLT2i in patients with symptomatic chronic HFrEF to reduce HHF and CV mortality, irrespective of the presence of T2D. More recently, empagliflozin now has FDA approval for reducing HHF or CV death among patients with HFpEF, defined as left ventricular EF being greater than 40%, with or without diabetes^7^. With the comprehensive management of cardio-renal-metabolism being emphasized, the utilization of SGLT2i is expected to increase over the years, not only in patients with T2D but also in those with CV disease or diabetic kidney disease.

Regarding diabetic ketoacidosis (DKA) related to SGLT2i, these medications have been associated with an elevated risk of DKA^8,9^. In 2015, the FDA issued a warning about the potential for these drugs to cause ketoacidosis, which generated considerable attention^10^. Numerous studies have documented instances of 'euglycemic' DKA (EDKA), generally characterized by normoglycemia with a plasma glucose level of less than 200 mg/dL, in T2D patients treated SGLT2i^11–14^.

In the perioperative setting, diagnosing SGLT2i-related EDKA becomes more complicated due to the specific postoperative status of patients. Several studies have already highlighted the issue of unrecognized diagnosis of SGLT2i-related EDKA in this context. Thiruvenkatarajan. et al. reported a 89% prevalence of postoperative SGLT2i-related EDKA in systematic review^15^, while Blau. et al. found a 71% incidence in their research using FDA data^16^. Among cardiac surgery patients, Murugesan, K.B. observed a 70.8% incidence of SGLT2i-related ketoacidosis (KA)^17^. Given these findings, it is imperative to differentiate SGLT2i-induced KA in cardiac surgery patients for the following reasons:

1. Due to shared patient population with HF, cardiac surgery patients show a higher incidence of SGLT2i usage than other surgical patients.
2. While the surgical stress is a known risk factor for SGLT2i-induced EDKA, cardiac surgery is unique in the implementation of cardiopulmonary bypass (CPB), setting it apart from other surgical procedures.
3. The laboratory presentations of SGLT2i-induced KA may mimic those of other severe postoperative complications in cardiac surgery patients, leading to potential diagnostic delays and additional expenditures.

Moreover, the clinical application of SGLT2i expands in CV patients, six cases of postoperative SGLT2i-related KA following cardiac surgery were identified in our center. Given the subtle presentation of SGLT2i-related KA, we hypothesized that it might be underdiagnosed, prompting a retrospective analysis of cardiac surgery patients who were preoperatively administered SGLT2i. Due to the retrospective nature of the study, confirmation of ketone body presence could not be conducted for all patients except the six identified; hence, their condition was termed as SGLT2i-related metabolic acidosis (MA).

The primary aim of this study is to quantify the incidence of postoperative SGLT2i related KA or MA and to identify related risk factors. Such insights could assist clinicians in differentiating SGLT2i-associated postoperative KA or MA, which is typically non-life-threatening^18^, from cardiac or other severe causes of MA, thus preventing unnecessary tests and prolonged intensive care unit (ICU) stays. Furthermore, this study assessed the impact of CPB, which induces a reduction in plasma protein concentration during bypass, on SGLT2i-related KA or MA^19^.

## Materials and methods

**Figure 1:**
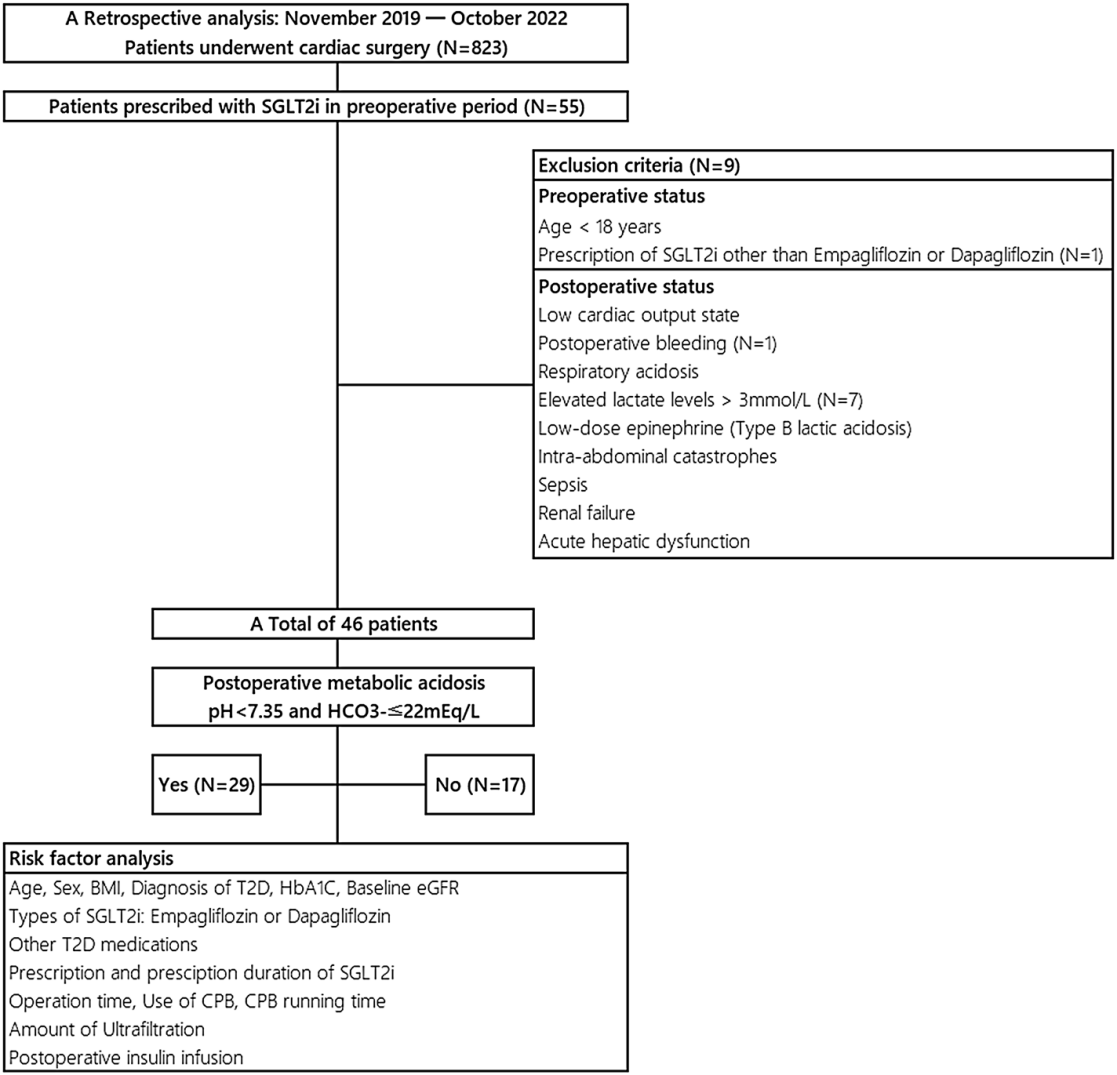
Patient selection, exclusion criteria and risk factor analysis. From November 2019 to October 2022, we conducted a retrospective analysis on 823 cardiac surgery patients at our center, 46 of whom were preoperatively treated with SGLT2i, specifically dapagliflozin or empagliflozin. Based on postoperative arterial blood gas analysis, patients were categorized into two groups: those with postoperative metabolic acidosis and those without. *SGLT2i* sodium-glucose transporter 2 inhibitors, *BMI* body mass index, *T2D* type 2 diabetes, *HbA1c* Hemoglobin A1C, *eGFR* estimated glomerular filtration rate, *CPB* cardiopulmonary bypass.

This study is a retrospective analysis of 823 patients who underwent cardiac surgery at Incheon Sejong Hospital from November 2019 to October 2022. Of the initial cohort, 55 were preoperatively prescribed SGLT2i. Exclusion criteria were set for conditions likely to induce postoperative acidosis^20^, including low cardiac output, postoperative bleeding, respiratory acidosis, elevated lactate levels (> 3 mmol/L), low-dose epinephrine (Type B lactic acidosis), intra-abdominal catastrophes, sepsis, renal failure, and acute hepatic dysfunction. An additional patient prescribed an SGLT2i other than Empagliflozin or Dapagliflozin was excluded for study consistency. Subsequently, the refined cohort consisted of 46 patients, stratified into two categories based on postoperative laboratory findings: the MA group (pH < 7.35 and HCO3- ≤ 22 mEq/L, n = 27) and the non-MA group (pH ≥ 7.35 or HCO3- > 22 mEq/L, n = 19). [Fig. 1] In both groups, we collected data on preoperative baseline characteristics, intraoperative factors, and immediate postoperative factors to identify risk factors associated with SGLT2i-related KA or MA.

To clarify, the postoperative period generally refers to the time starting immediately after surgery, typically beginning with the patient's admission to the ICU. At our institution, preoperative glycemic control in T2D patients is managed with a glucose-insulin mix or glucose-insulin-potassium (GIK) solution, following endocrinologist consultation. Postoperatively, we follow ICU protocol to maintain blood glucose between 110 and 150 mg/dL using insulin infusion^20^. Both preoperative and postoperative insulin infusions are administered as mentioned earlier.

### Statistical analysis

The IBM SPSS software, version 21.0, was utilized for data analysis. Continuous variables were expressed as mean ± standard deviation (SD) or as median (25th and 75th percentiles), while categorical variables were presented as frequency counts and percentages (n%). Independent t-tests and χ^2^ tests were employed, as appropriate, to compare variables such as types of SGLT2i, arterial blood gas analyses (ABGA), duration of mechanical ventilator (MV) support, and length of ICU stay between the postoperative MA group and the non-MA group. The Mann–Whitney U test was used to compare the number of inotropes administered between the two groups. Univariate logistic regression analysis was conducted to identify potential associated risk factors in preoperative, intraoperative and postoperative period. The covariables for the multivariable logistic regression model were selected based on clinical judgement and a comprehensive review of existing literature. The variables evaluated in this model include Body Mass Index (BMI), the status of preoperative insulin usage, duration of SGLT2i prescription and cessation, operating time, CPB duration, and postoperative insulin utilization. Also, CPB running time was divided into before and after 60 min to indicate a short CPB running time. While there is no basis for specifically choosing 60 min, but generally, one shot of cardioplegia lasts between 60 to 90 min, depending on the type of cardioplegia used. We set 60 min as the criterion for short CPB time, which is also related to the reduction of plasma protein concentration and the duration or amount of ultrafiltration. The impact CPB duration on postoperative MA or KA associated with SGLT2i was examined in a subgroup analysis of patients with sufficient prescription duration. The odds ratio (OR) or hazard ratio (HR) are presented with a 95% confidence interval(CI) for inferential statistics, and a two-sided P-value of less than 0.05 was statistically significant.

### Ethics approval and consent to participate

Given the retrospective design of this study and the fact that there was no harm to participants, the Institutional Review Board of Incheon Sejong Hospital approved a waiver for the requirement of informed consent. (IRB approval no. 2022-09-001). All personal and clinical data, including laboratory results and patient outcomes, were obtained from the patients' medical records. All the methods were carried out in accordance with relevant guidelines and regulations.

## Results

### Demographic and postoperative profile of the study cohort

In a retrospective study of 46 patients undergoing cardiac surgeries, 26 (56.5%) were prescribed dapagliflozin, and 29 (63%) developed postoperative SGLT2i-related MA. Clinical and demographic characteristics of the study population are shown in [Table 1]. The average of age was 62.2 ± 11.14 year and BMI was 24.78 ± 3.3 kg/${m}^{2}$. Of the 46 patients, 33 (71.3%) were men, and 42 (91.3%) had a past medical history of T2D. Blood glucose levels in both groups were investigated, and no statistical difference was found (117.7 ± 61.2 vs. 191.4 ± 35.3; p = 0.373). Furthermore, no statistically significant difference was noted between the two groups regarding the duration of MV support or the length of ICU stay. When evaluating the quantity of inotropes administered, instances of postoperative MA associated with SGLT2i exhibited a greater proportion of patients with a high inotropic demand (more than 2 agents); however, this variance was not statistically significant. (5 (17.2%) vs. 0 (0%); p = 0.140).

**Table 1:** Basic characteristics of SGLT2i-related postoperative metabolic acidosis.

Characteristics			
Age (years)	62.2 ± 11.14		
Sex			
Male	33 (71.3)		
Female	13 (28.3)		
BMI (kg/m^2^)	24.78 ± 3.3		
T2D diagnosis			
Yes	42 (91.3)		
No	4 (8.7)		
HbA1C (%)	7.69 ± 1.4		
Other T2D medications			
None	8 (17.4)		
OHA	33 (71.7)		
Insulin + OHA	5 (10.9)		
eGFR (mL/min/1.73 m^2^)	88.5 ± 15.7		
Operation			
CABG	28 (60.9)		
VALVE	9 (19.6)		
VALVE + CABG	2 (4.3)		
LVAD	6 (13)		
OTHERS	1 (2.2)		

	Postoperative metabolic acidosis		p value
	Yes	No	
SGLT2i			0.81
Dapagliflozin	16(55.2)	10(58.8)	
Empagliflozin	13(44.8)	7(41.2)	
Arterial blood gas analysis			
pH	7.29 ± 0.03	7.4 ± 0.05	< 0.001*
Bicarbonate (mEq/L)	18.3 ± 2.5	21.6 ± 2.5	< 0.001*
Base Excess (mEq/L)	-7.5 ± 2.8	-3.67 ± 6.1	< 0.001*
Anion gap (mEq/L)	10.9 ± 2.9	7.53 ± 3.1	0.03*
Blood glucose level (mg/dL)	117.7 ± 61.2	191.4 ± 35.3	0.373
Lactate (mmol/L)	1.29 ± 0.61	1.07 ± 0.53	0.241
Serum albumin (g/dL)	2.8 ± 0.3	2.9 ± 0.2	0.183
The number of Inotropes			0.14
0–1	24 (82.8)	17 (100)	
≥ 2	5 (17.2)	0 (0)	
Duration of MV support (hours)	5.24 (3.85, 6.5)	5.28 (4.13, 6.42)	0.92
ICU stay (hours)	23.66 (21, 26.3)	22.2 (18.2, 26.1)	0.92

This table outlines the characteristics of 46 postoperative metabolic acidosis related to SGLT2i. The average age was 62.2 ± 11.14 years, and BMI was 24.78 ± 3.3 kg/m^2^. Most patients were men (71.3%) with a history of T2D (91.3%). They were treated with either dapagliflozin (56.6%) or empagliflozin (43.4%), showing no significant difference in blood glucose levels or ICU stay duration. A slightly higher, yet statistically insignificant, inotropic demand was observed in a subset of patients (p = 0.140). Reference ranges: eGFR (85–116 mL/min/1.73 m^2^); pH (7.35–7.45); HCO3- (22–26 mEq/L); Base excess/deficit (− 4– + 2 mEq/L); Anion gap (4–12 mEq/L); Blood glucose level (70-140 mg/dL); Lactate (0.5–2.2 mmol/L).

*SGLT2i* sodium-glucose transporter 2 inhibitors, *BMI* body mass index, *T2D* type 2 diabetes, *HbA1c* Hemoglobin A1C, *OHA* oral hypoglycemia agents, *eGFR* estimated glomerular filtration rate, *CABG* coronary artery bypass grafting, *LVAD* left ventricular assist device, *MV* mechanical ventilator, *ICU* Intensive care unit.

### Clinical variables and incidence of SGLT2i -related postoperative MA

The potential risk factors, preoperative, intraoperative and postoperative variables, associated with SGLT2i-related postoperative MA were examined in univariate and multivariable regression analyses [Table 2]. We included factors with a p-value of less than 0.2 in the univariate analysis as significant variables in the multivariable logistic regression test. Additionally, we incorporated the presence of insulin-dependent T2D, a previously known precipitating factor for DKA, and the cessation time of more than 72 h for SGLT2i, as specified in the guidelines for the prevention of EDKA, into the analysis.

**Table 2:** Risk factor analysis of SGTL2i-related postoperative metabolic acidosis in post cardiac surgery patients.

Characteristics	Postoperative metabolic acidosis		Univariate logistic regression		Multivariable logistic regression	
	Yes	No	Unadjusted OR (95% CI)	p value	Adjusted OR (95% CI)	p value
Preoperative	(n, 29)	(n, 17)				
Age	62.1 ± 11.5	62.3 ± 10.8	1.00 (0.94–1.05)	0.941		
Sex			0.91 (0.25–3.61)	0.894		
Male	21 (72.4)	12 (70.6)				
Female	8 (27.6)	5 (29.4)				
BMI (kg/m^2^)	24.1 ± 2.8	25.8 ± 3.9	0.85 (0.68–1.03)	0.105	0.81 (0.63–1.04)	0.106
T2D			–	0.993		
Yes	25 (86.2)	17 (100)				
No	4 (13.8)	0 (0)				
HbA1C (%)	7.6 ± 1.5	7.7 ± 1.4	0.96 (0.63–1.46)	0.836		
Preoperative glucose-insulin mixed infusion			1.56 (0.44–5.47)	0.487		
Yes	20(68)	10(58.8)				
No	9(31)	7(41.2)				
Other T2D medications			0.47 (0.14–1.60)	0.231	0.38 (0.06–2.23)	0.288
No	6(20.7)	2(11.8)				
Other OHA	21(72.4)	12(70.6)				
OHA + Insulin	2(6.9)	3(17.6)				
Prescription duration of SGLT2i			11.7 (1.65–237)	0.032*	31.58 (1.97–505.23)	0.014*
> 7 days	28(96.6)	12(70.6)				
≤ 7 days	1(3.4)	5(29.4)				
Cessation duration of SGLT2i			0.58 (0.16–2.20)	0.42	0.30 (0.05–1.81)	0.19
> 72 h	7(24.1)	6(35.3)				
≤ 72 h	22(75.9)	11(64.7)				
eGFR (mL/min/1.73 m2)	90 ± 13.6	86 ± 18.9	1.02 (0.98–1.06)	0.409		
Intraoperative						
Operation			1.06 (0.62–1.81)	0.803		
CABG	18(62.1)	10(58.8)				
Valve	5(17.2)	4(23.5)				
Valve + CABG	1(3.4)	1(5.9)				
LVAD	4(13.8)	2(11.8)				
Others	1(3.4)	0(0)				
Operation time (min)	260.8(230.9, 290.8)	307.9 (252.6, 363.2)	0.99 (0.99–1.00)	0.102	0.998 (0.98–1.00)	0.81
Use of CPB			0.49 (0.14–1.65)	0.256		
Yes	12(41.4)	10(58.8)				
No	17(58.6)	7(41.2)				
CPB time (min)	45.8(21.2, 70.4)	77.8(38, 117.6)	0.99 (0.98–1.00)	0.14		
CPB 60			0.40 (0.11–1.37)	0.142	0.50 (0.09–2.76)	0.427
> 60 min	9 (31.0)	9 (52.9)				
≤ 60 min	20 (69.0)	8 (47.1)				
Ultrafiltration (cc)	1603 (0, 3892)	2241 (0, 4555)	0	0.361		
Postoperative						
Insulin infusion			0.44 (0.12–1.49)	0.197	0.40 (0.07–2.22)	0.298
Yes	13(44.8)	11(64.7)				
No	16(55.2)	6(35.3)				

The table summarizes perioperative risk factors related to SGLT2i-related postoperative metabolic acidosis evaluated through univariate and multivariable regression analyses. A prescription duration exceeding one week was notably associated with this acidosis (Unadjusted OR, 11.7; 95% CI 1.65–237; p = 0.032*), and this remained significant in multivariable analysis (adjusted OR, 31.58; 95% CI 1.97–505.23; p = 0.014*).

*SGLT2i* sodium-glucose transporter 2 inhibitors, *BMI* body mass index, *T2D* type 2 diabetes, *HbA1c* Hemoglobin A1C, *OHA* oral hypoglycemia agents, *eGFR* estimated glomerular filtration rate, *CABG* coronary artery bypass grafting, *LVAD* left ventricular assist device, *CPB* cardiopulmonary bypass, *OR* odds ratio, *CI* confidence interval.

### Preoperative factors

In the unadjusted analysis, several factors—including age, sex, BMI, premedical history of T2D, hemoglobin A1C (HbA1c) levels, preoperative glucose-insulin mixed infusion, use of other T2D medications, duration of SGLT2i cessation, and baseline estimated glomerular filtration rate (eGFR)—did not exhibit statistically significant differences between the postoperative MA group and the non-MA group. However, a prescription duration of SGLT2i of more than one week was associated with SGLT2i-related postoperative MA (OR, 11.7; 95% CI 1.65–237; p = 0.032*). In the multivariable analysis, this association remained significant (adjusted OR, 31.58; 95% CI 1.97–505.23; p = 0.014*).

### Intraoperative factors

Regarding intraoperative factors, we assessed the types of operations conducted, the duration of these operations, the utilization of CPB, the duration of CPB running time, instances where CPB exceeded 60 min (referred as CPB 60), and the volume of ultrafiltration. In the univariate analysis, no statistically significant differences in intraoperative factors between the two groups were observed. In the adjusted model, which examined potential risk factors associated with postoperative MA or KA related to SGLT2i—including operation time and CPB 60—no statistical significance was found.

### Postoperative factors

In both the univariate and multivariable models, the immediate postoperative administration of insulin infusion did not show any statistically significant differences between the group with postoperative MA related to SGLT2i and the non-MA group. (Unadjusted OR: 0.44; 95% CI 0.12–1.49; p = 0.197; Adjusted OR: 0.40; 95% CI 0.07–2.22; p = 0.298).

### Subgroup analysis: impact of CPB running time on SGLT2i-related postoperative MA in patients with sufficient prescription duration

A subgroup analysis was conducted to assess the impact of CPB duration in patients with a sufficient SGLT2i prescription duration over 7 days (N = 40). Table 3 shows that a CPB duration ≤ 60 min is associated with a significantly higher risk of SGLT2i-related postoperative MA (HR 2.94; 95% CI 0.50–5.38, p = 0.018*).

**Table 3:** Subgroup Analysis: Impact of CPB Running Time on SGLT2i-Related Postoperative Metabolic Acidosis in patients with sufficient Prescription Duration.

	CPB duration ≤ 60 min	p value	CPB duration > 60 min	p value
	HR (95% CI)		HR (95% CI)	
Prescription duration				
> 7 days	2.94 (0.50–5.38)	0.018*	–	0.994

A subgroup analysis was conducted to assess the impact of CPB duration in patients with a sufficient SGLT2i prescription duration over 7 days (N = 40). The table shows that a CPB duration ≤ 60 min is associated with a significantly higher risk of SGLT2i-related postoperative metabolic acidosis(HR 2.94; 95% CI 0.50–5.38, p = 0.018*).

*SGLT2i* sodium-glucose transporter 2 inhibitors, *CPB* cardiopulmonary bypass, *HR* hazard ratio, *CI* confidence interval.

## Discussion

For individuals with T2D, the prevalence of DKA or EDKA following the introduction of SGLT2i remains uncertain. In predominantly non-operative settings, in clinical trials involving SGLT2i for T2D treatment, the incidence of DKA ranged from 0.2 to 0.8 cases per 1000 patient-years^21,22^. The DKA incidence in T2D patients on SGLT2i might not be higher than in the wider diabetic population; however, clinical trials may not fully reflect real-world incidences. In a meta-analysis of 36 trials that evaluated T2D drug therapies, SGLT2i were the only class of drugs associated with an increased risk of DKA compared to other therapies^23^. A large-scale study from Canada and the United Kingdom, involving over 350,000 patients and 500 DKA cases, noted an increased risk with SGLT2i (dapagliflozin, empagliflozin, canagliflozin) compared to dipeptidyl peptidase-4 inhibitors, with respective incidence rates of 2.03 and 0.75 per 1,000 patient-years, and heightened DKA risks associated with dapagliflozin (HR: 1.86) and empagliflozin (HR: 2.52)^24^.

As known, several factors are recognized to precipitate DKA in the context of SGLT2i use. Patient characteristics such as insulin deficiency, commonly seen in latent autoimmune diabetes in adults, type 1 diabetes, or some individuals with long-standing T2D, have been noted^25^. Metabolic stressors like surgical interventions, vigorous exercise, myocardial infarctions, strokes, severe infections, and prolonged fasting have also been identified as significant contributors^25,26^. Other risk variables that could potentially predispose an individual to KA include pancreatic insulin deficiency, reductions in prescribed insulin dosage, caloric restriction, alcohol misuse and acute febrile illness.

Regarding perioperative patients with SGLT2i-related KA or MA, Thiruvenkatarajan et al. reported a prevalence of 89% for postoperative SGLT2i-related EDKA in their systematic review^15^. Blau. et al. using FDA data, found a comparable incidence rate of 71% (29 out of 51)^16^ and Murugesan, K.B. observed a 70.8% (17 out of 24) for SGLT2i-associated EDKA in cardiac surgery patients^17^. Each study has different criteria, making comparison somewhat nonsensical; however, our study identified a 63% incidence (29 out of 46) of SGLT2i-related KA or MA, which aligns with previous studies and shows no significant statistical difference, as evidenced by p-values of 0.598 and 0.278, respectively.

Patients undergoing cardiac surgery represent a unique demographic at an intersection of heightened risk for postoperative MA or KA associated with SGLT2i, primarily because they have pre-existing CV disease, which often necessitates the prescription of SGLT2i. Moreover, these patients typically undergo fasting and experience the surgical stress. Despite these compounded risk factors, the risk-to-benefit ratio overwhelmingly supports the continued use of SGLT2i for managing CV disease or T2D, and current guidelines uphold this stance.

With the rising use of SGLT2i for treating CV disease, we observed six patients who developed postoperative KA associated with these medications. Table 4 details these patients with confirmed ketone bodies who suffered from SGLT2i-related postoperative KA. Notably, there was no consistent pattern regarding the hours of onset of ketoacidosis among these patients. For our first patient (case #1) was involved in extended diagnostic process such as bedside transthoracic echocardiography and cardiac angiography and resulting in a 24-h diagnostic delay. This initial experience facilitated more straightforward diagnoses in subsequent cases, highlighting the importance of considering SGLT2i related EDKA in the care of post-cardiac surgery patients to prevent diagnostic delays. Interestingly, one patient (case #6) without a prior medical history of T2D also exhibited postoperative KA. This implies that these incidents may not be limited only to patients with diabetes, but might also include those using SGLT2i. Therefore, we referred to this condition not as SGLT2i related EDKA, but as postoperative MA or KA related to SGLT2i, omitting the term 'diabetic’. A mechanism associated with SGLT2 inhibition appears to induce an early transition to fat metabolism, leading to an accumulation of ketones and resulting in KA or MA^27^.

**Table 4:** Clinical characteristics of SGLT2i-related postoperative ketoacidosis cases.

Characteristics	1	2	3	4	5	6
Preoperative						
Age (years)	59	59	61	73	58	62
BMI (kg/m^2^)	24.6	24.8	22.7	25.5	23.4	23.6
Sex	M	F	M	F	M	F
History of T2D diagnosis	10 years	10 years	20 years	27 years	7 years	N
HbA1C (%)	7.5	7.3	8.8	10.3	6.8	5.9
SGLT2i	Empagliflozin	Empagliflozin	Empagliflozin	Empagliflozin	Empagliflozin	Dapagliflozin
Prescription duration of SGLT2i	8 days	16 months	12 months	12 months	15 months	12 months
Duration of SGLT2i cessation (hours)	48	36	48	72	24	120
Other T2D medications	Other OHA	Other OHA	Other OHA	Other OHA	Other OHA	None
Preoperative glucose-insulin mixed infusion	N	N	Y	Y	N	N
eGFR (mL/min/1.73 m2)	102	93	101	70	95	98
Intraoperative						
Operation	CABG	MVR, MAZE	CABG	CABG	CABG	LVAD
Operation time (min)	240	240	275	285	230	120
Use of CPB	N	Y	N	N	N	Y
CPB time (min)	0	120	0	0	0	47
Ultrafiltration (cc)	0	4500	0	0	0	1800
Postoperative						
Arterial blood gas analysis						
Arterial pH	7.21	7.23	7.27	7.29	7.25	7.27
Bicarbonate (mEq/L)	14.2	13.6	13.6	16.8	17.2	17.1
Base Excess (mEq/L)	− 12.7	− 8.7	− 10.8	− 12.6	− 9.2	− 9.2
Anion gap (mEq/L)	15.8	15	10	6.2	10.5	12.9
Blood glucose level(mg/dL)	122	126	95	117	120	297
Lactate (mmol/L)	1.2	0.8	0.5	0.5	0.9	2.5
Urine ketone	3 +	3 +	3 +	2 + 30	3 +	1 +
Total plasma ketone (umol/L)	7635.8	3522.1	4868.3	3165.3	7753.4	3309.9
Time to onset from operation (hours)	9	15	3	18	2	5
Time to diagnosis from onset (hours)	24	0	0	6	0	0
Time to recovery from intervention (hours)	3	5	3	2	8	1
The number of Inotropes	0	0	2	0	0	1
Duration of MV support (hours)	8	4	8	4	4	9
ICU stay (hours)	42	20	23	20	22	16

The details of cases involving SGLT2i-related postoperative KA are outlined. Case #1, as the first case, experienced a 24-h diagnostic delay due to extensive tests. Notably, case #6, who had no history of T2D, also manifested KA, suggesting that these incidents might not be confined to diabetic patients. Reference ranges: eGFR (85–116 mL/min/1.73 m2); pH (7.35–7.45); HCO3- (22–26 mEq/L); Base excess/deficit (− 4- + 2 mEq/L); Anion gap (4–12 mEq/L); Blood glucose level (70–140 mg/dL); Lactate (0.5–2.2 mmol/L); Total plasma ketone (0-600umol/L).

*SGLT2i* sodium-glucose transporter 2 inhibitors, *KA* ketoacidosis, *T2D* type 2 diabetes, *BMI* body mass index, *OHA* oral hypoglycemia agents, *HbA1c* Hemoglobin A1C, *eGFR* estimated glomerular filtration rate, *CPB* cardiopulmonary bypass, *MV* mechanical ventilator, *ICU* Intensive care unit, *CABG* coronary artery bypass grafting, *MVR* mitral valve replacement, *LVAD* left ventricular assist device.

We conducted a retrospective review to assess the incidence and risk factors of postoperative MA or KA related to SGLT2i in 46 cardiac surgery patients; 63% (29 patients) developed postoperative MA associated with SGLT2i [Fig. 1]. Although the threshold for MA might differ across clinical laboratories, we characterized SGLT2i-related postoperative MA based on the criteria of a pH < 7.35 and an HCO3- ≤ 22 mEq/L^28^. While our chosen threshold could be relatively liberal, we believe it is crucial to initially rule out benign but potentially concerning laboratory anomalies in post-cardiac surgery patients.

Cessation duration, according to the prescribing information^29,30^, dictates that SGLT2i should be discontinued 72 h prior to surgery.. In the univariate analysis, a cessation duration of more than 72 h in the preoperative period did not result in a significant decrease in the occurrence of SGLT2i-related postoperative MA or KA (unadjusted OR, 0.58; 95% CI 0.16–2.20; p = 0.42) [Table 2]. This lack of significance might suggest that the advised cessation period is shorter than actually needed.

A significant proportion of the cohort, 12 out of 46 (26%), were prescribed SGLT2i at the time of their CV diagnosis, within 1 month before their cardiac surgery. The univariate analysis indicated that a prescription duration exceeding 7 days significantly increased the incidence of SGLT2i-related postoperative MA (unadjusted OR, 11.7; 95% CI 1.65–237; p = 0.032*). This significance persisted in the multivariable analysis (adjusted OR, 31.58; 95% CI 1.97–505.23; p = 0.014*) [Table 2]. Our assumption is that the shift to fat metabolism induced by SGLT2 inhibition takes at least a few days, and patients who were prescribed for less than 7 days actually took SGLT2i for 3–5 days, considering the preoperative cessation period.

Furthermore, a subgroup analysis was performed to assess the impact of CPB running time on the occurrence of SGLT2i-related postoperative MA or KA. [Table 3] While ultrafiltration is employed to concentrate blood during CPB operations, reductions in both plasma colloid osmotic pressure and total protein concentration were noted following CPB use^19^. Following a decrease in protein levels, including drug-binding proteins, we hypothesized that the utilization of CPB might reduce the incidence of postoperative MA or KA associated with SGLT2i. Among patients prescribed SGLT2i for a sufficient duration (> 7 days, N = 40), those undergoing cardiac surgery with a short CPB running time (≤ 60 min) exhibited a higher risk of postoperative SGLT2i-related MA(OR, 2.94; 95% CI 0.50–5.38; p = 0.018*).

Rapid resolution of metabolic abnormalities induced by SGLT2i was observed after initiating insulin and dextrose infusions, as stated by Aaron Lau et al. in their case series^31^. Our findings were consistent with this; resolution of postoperative KA related to SGLT2i was easily achieved in our cases as well [Table 4]. Given that the ICU hyperglycemic management protocol for cardiac surgery patients targets a postoperative blood sugar range of 110 to 150 mg/dL and initiates a corrective protocol for levels above 150 mg/dL^20^, it was hypothesized that this protocol could incidentally manage undiagnosed cases of postoperative MA or KA related to SGLT2i. Despite this assumption, our analysis found that postoperative insulin infusion did not significantly affect the incidence of MA associated with SGLT2i (unadjusted OR, 0.44; 95% CI 0.12–1.49; p = 0.197; adjusted OR, 0.40; 95% CI 0.07–2.22; p = 0.298). [Table 2].

For our strategy in managing post-cardiac surgery patients without kidney disease, we utilized a GIK solution to treat KA associated with SGLT2i. Electrolyte imbalances in post-cardiac surgery patients can provoke arrhythmias, making the restoration of potassium levels especially crucial in this patient population.

Based on our findings, we advocate for the verification of ketone bodies using urine or serum ketone tests in post-cardiac surgery patients who present with high anion-gap MA, a condition solely attributable to the preoperative use of SGLT2i. As a time and cost-effective approach, we assess urine ketone levels through routine urinalysis, which provides results within 30 min in these patients. When patients present with a urine ketone level exceeding 2 +, we subsequently conduct a serum ketone test, which often requires 2–3 days for results, and initiate GIK solution administration concomitantly.

## Limitations

Given the nature of a retrospective observational study, ketone body tests were not routinely included in postoperative laboratory tests, so ketone body confirmations could not be conducted for all patients. Regarding ICU management, there was a lack of uniformity among physicians, and unnecessary injections of sodium bicarbonate affected anion gap and pH levels. Furthermore, this issue spoiled anion gap, which is crucial for differentiating postoperative hyperchloremic metabolic acidosis, thereby preventing the establishment of an accurate exclusion criteria. As a single-center study, there were deviations in operation entities, with a scarcity of aortic surgeries.

## Conclusion

The CV benefits of SGLT2i in patients with CV disease are undeniably profound. Not every case of KA is necessarily harmful, but the FDA-issued warnings about these risks have not been sufficiently integrated into perioperative care protocols. The lack of awareness complicates diagnosis and treatment of postoperative metabolic issues related to SGLT2i. However, our research shows that timely diagnosis and proper treatment can ease the patient's transition from ICU support to general wards.

In describing the postoperative metabolic issues observed in our study, we chose not to use the term 'diabetes' to highlight the possibility that these conditions might extend beyond patients with T2D. Further research is needed concerning the optimal duration for preoperative cessation of SGLT2i. Additionally, it is imperative to study the effects of CPB institution on SGLT2i-related postoperative KA in patients, including the assessment of SGLT2i-induced KA among nondiabetic individuals.

## Data Availability

The datasets generated during and/or analyzed during the current study are available from the corresponding author on reasonable request.

## Abbreviations

FDA: Food and Drug administration
SGLT2i: Sodium-glucose transporter 2 inhibitors
T2D: Type 2 diabetes
CV: Cardiovascular
HFrEF: Heart failure with reduced ejection fraction
HFpEF: Heart failure with preserved ejection fraction
HFmrEF: Heart failure with mildly reduced ejection fraction
HHF: Hospitalization for heart failure
DKA: Diabetic ketoacidosis
EDKA: Euglycemic diabetic ketoacidosis
KA: Ketoacidosis
CPB: Cardiopulmonary bypass
MA: Metabolic acidosis
ICU: Intensive care unit
GIK solution: Glucose-insulin-potassium solution
SD: Standard deviation
ABGA: Arterial blood gas analyses
MV: Mechanical ventilator
BMI: Body mass index
OR: Odds ratio
HR: Hazard ratio
CI: Confidence interval
HbA1c: Hemoglobin A1C
eGFR: Estimated glomerular filtration rate
